# A Day in Bed

**Published:** 1888-06-23

**Authors:** 


### THE DOCTOR'S PULPIT.

### A DAY IN BED.

THE medicine of work has already been recommended from the Doctor's Pulpit; but, with the approach of the holiday season, a sermon may fitly be preached on the medicine of rest. There are many ways of resting, as there are many reposeful attitudes, as different as the varieties of temperament that incline us towards them. The occupant of the Pulpit this week cannot speak of all these; he ventures only to say a word for one particular dose of the medicine of rest, which is not only neglected, but often maligned.

Occasionally at our railway stations a placard may be seen announcing that a certain spot—suburban palace of delight or seaside village (there and back half-a-crown)—is "the place to spend a happy day." The preacher adds one more to these numerous suggestions, and tells his congregation that a very suitable place to spend a happy day—and cheaply, too—is in bed. He is aware that a strong prejudice exists against this form of recreation, and he is prepared to have a good many of his readers shocked at the outset, but he begs them to pause a little before they condemn his prescription untried. He does not recommend it to everybody. There are plenty of people who don't habitually do one day's honest work in the week, and then complain of insomnia, and want narcotics and sedatives of all kinds; they don't need a day in bed, or any more opportunities of resting than they already have; on the contrary, enough hard physical work, or even mental worry to shake them out of their self-conscious egoism, would do them all the good in the world. But if there are people who work too little, there are a large number who work far too much, who get so much into the habit of working that they make even their holidays more laborious than restful. To them is recommended a day in bed.

Every living creature, every living organism, wants rest in very generous proportion to work. The human heart, usually supposed to be an organ that never rests till death stops its motion, is, as a matter of fact, working only a third of the time; the remaining two-thirds is spent in resting between the beats. Of course this resting-time is absolutely short, but in proportion to the working time it is relative large; and the ratio of one-third work to two-thirds rest shows us that it is a wise instinct that has led us to assign as the average period of labour eight hours a-day, with eight hours for sleep, and eight hours for eating and recreation. Some people, of course, will tell you that they do not require eight hours' sleep; seven, six, five are enough for them. When any one says that you may be sure of one of two things, either that the speaker counts only that sleep which he takes in bed, forgetting those delicious scraps of slumber he indulges in during the day—the pleasant "forty winks" in an easy chair, and the "resting the eyelids" over book or newspaper; or that he is on the high road to a premature breakdown unless some benignant illness steps in and forces him to take the necessary rest. *Qui dort dine,* says the French proverb; sleep is a food—that is, a means of repairing tissue-waste, and to stint oneself in it is every bit as foolish and suicidal as to go without the necessary rations of bread and beef.

It may be thought that the doctor is preaching on sleep in general without much connexion with the avowed subject of his discourse—a day in bed. Not at all; he only wants to impress on his congregation the general fact that sleep, and plenty of sleep, is a necessary of life, and not, as many of them seemed to think, a bad habit.

But there are many people who cannot get a sufficient daily allowance of sleep; their work does not permit of it; others there are again, brain-workers, whose toil takes more out of their system than the ordinary allowance of sleep will repair. These require an occasional supplementary allowance of rest—a day in bed. If you find yourself getting tired at mid-day and absent-minded over your work, however much you bully your brain to keep fixed on it; if you begin to forget your engagements and to feel each new task imposed on you a burden like the proverbial last straw; if you are beginning to cherish an irritable conviction that your wife neglects your comfort and your subordinates neglect your interests, then you may be sure that your nervous system is over-strained, and that you want to relax and recruit it by a day in bed. The preacher is well aware that, unless bitter experience has made you wise, you won't take it. You will conclude that you want exercise, and so by way of resting one set of nerves you will begin to tire another. A true-born Briton hates laziness, and if he suspects himself of a desire to lounge he tortures himself into a feverish activity.

Everybody believes exercise to be good for them. Yes, it is very good, absolutely essential, when you are in a fit state to undertake it, but that is not when you have already exhausted all your store of energy. Then you want rest, at first as absolute as you can get it, then modified by a gradually increasing activity. At the end of a day or two of idleness you may, without injury, with absolute profit to your whole nature, indulge in that hill-climbing, that bicycle ride, that tennis tournament, which, undertaken the day after you left your ordinary routine of work, might possibly strain your heart, over-tax your lungs, and wear out your nerves. No; when you feel thoroughly done up, cross, and exhausted, don't go and spur up poor over-worked, over-tired nature; it is a cruelty, and you can hardly blame her if, struggling along however bravely to do a task she is not fit for, she one day stumbles and throws you. Try a day in bed as a preliminary if you have a few days' holiday before you; if but one scant twenty-four hours before you must begin work again, you may do worse than spend it all in complete rest. A certain woman who had led a life of great activity and responsibility attributed the admirable health she enjoyed at the age of eighty to the fact that she had habitually spent a day in bed every week. This is more than most of us want; a day every month or six weeks will serve to keep us well and cheerful. And it need not be spent drearily. A novel—not the "book of the season" that everyone is talking of and you must read, but a good old-fashioned romance, by preference, one which you have read before, so that you know what to skip, will help to make the time pass pleasantly between your dreams, and you may be quite sure of having all your womenkind crowding round you with dainties and caresses. All who try it will find, besides the good it will do them, there is a great deal to be said for a day in bed.

